# Enhancer Recognition: A Transformer Encoder-Based Method with WGAN-GP for Data Augmentation

**DOI:** 10.3390/ijms242417548

**Published:** 2023-12-16

**Authors:** Tianyu Feng, Tao Hu, Wenyu Liu, Yang Zhang

**Affiliations:** 1 College of Information Science & Engineering, Lanzhou University, Lanzhou 730000, China; 220220941641@lzu.edu.cn (T.F.); thu21@lzu.edu.cn (T.H.); 2 College of Ecology, Lanzhou University, Lanzhou 730000, China; liuwy19@lzu.edu.cn; 3 Supercomputer Center, Lanzhou University, Lanzhou 730000, China

**Keywords:** enhancer, deep learning, transformer, generative adversarial network

## Abstract

Enhancers are located upstream or downstream of key deoxyribonucleic acid (DNA) sequences in genes and can adjust the transcription activity of neighboring genes. Identifying enhancers and determining their functions are important for understanding gene regulatory networks and expression regulatory mechanisms. However, traditional enhancer recognition relies on manual feature engineering, which is time-consuming and labor-intensive, making it difficult to perform large-scale recognition analysis. In addition, if the original dataset is too small, there is a risk of overfitting. In recent years, emerging methods, such as deep learning, have provided new insights for enhancing identification. However, these methods also present certain challenges. Deep learning models typically require a large amount of high-quality data, and data acquisition demands considerable time and resources. To address these challenges, in this paper, we propose a data-augmentation method based on generative adversarial networks to solve the problem of small datasets. Moreover, we used regularization methods such as weight decay to improve the generalizability of the model and alleviate overfitting. The Transformer encoder was used as the main component to capture the complex relationships and dependencies in enhancer sequences. The encoding layer was designed based on the principle of k-mers to preserve more information from the original DNA sequence. Compared with existing methods, the proposed approach made significant progress in enhancing the accuracy and strength of enhancer identification and prediction, demonstrating the effectiveness of the proposed method. This paper provides valuable insights for enhancer analysis and is of great significance for understanding gene regulatory mechanisms and studying disease correlations.

## 1. Introduction

Enhancers [1] are noncoding deoxyribonucleic acid (DNA) sequences, which play a crucial role in regulating gene expression. They are responsible for controlling the timing and location of gene expression and play a key role in development and disease. Enhancers are highly dynamic and environment-dependent, responding to a wide range of signals and cellular contexts. For example, the genomes of humans and chimpanzees are approximately 98% similar [2,3,4]; however, there are numerous differences. Research has shown that the Forkhead Box P2 gene (FOXP2) [5] plays a crucial role in the development of language abilities. The FOXP2 enhancer in humans differs from that in chimpanzees, resulting in higher expression levels of the FOXP2 gene in specific regions of the human brain. This enhancer alteration is closely related to the evolution of human language ability. In recent years, with the advancement of genomics and epigenomics, the study of enhancers has become increasingly important, revealing the complexity of gene regulation and the role of noncoding DNA in diseases. Enhancer research has a wide range of applications in developmental biology, tumor biology, and regenerative medicine. Understanding enhancer function and regulation is essential for advancing our knowledge of gene expression and its implications in health and disease [6,7].

Enhancers, as key components of DNA, are associated with specific genomic sites of transcription factors [8] and chromatin modification enzymes through complementary pairing between the structural domains of transcription factors and specific DNA sequences. Once bound to enhancers, transcription factors can enhance gene transcriptional activity through various mechanisms. Enhancers can promote the proximity of regulatory regions and gene promoters by altering the three-dimensional structure of chromatin. They facilitate interactions between regulatory regions and promoters, leading to the formation of physical contacts. These contacts can create stable DNA loop structures, which promote interactions between transcription factors in regulatory regions and gene promoters, thereby enhancing gene transcription.

Enhancers are typically located upstream or downstream of the target gene [9]. Due to the looping structures present in a chromosome, enhancers can establish contact with the target gene even over long distances. The function of enhancers may be influenced by complex factors such as upstream and downstream regulatory sequences [9], regulatory proteins, and chromatin states. Therefore, experimental validation is required to determine whether the DNA region has enhancer activity. Additionally, with advances in high-throughput sequencing technology, large-scale enhancer identification can be conducted. However, processing, analyzing, and interpreting large-scale data remain challenging tasks, which necessitate the utilization of bioinformatics methods [10] and computational tools to uncover the characteristics and functions of enhancers, presenting a challenge in accurately localizing enhancers, particularly within complex gene regulatory regions.

Early DNA-sequencing methods were limited to sequencing short segments of DNA, which resulted in slow and inefficient sequencing with high costs. Consequently, our understanding of the genome remains limited. With the advent of high-throughput sequencing methods, researchers have been able to perform large-scale sequencing for various complex organisms, including humans. This has led to an increasing number of researchers investigating the regulatory structure of the human genome to develop therapeutic approaches for various diseases. Recent studies have linked nucleotide variations in enhancer-associated chromatin modifications to numerous phenotypic changes [11,12]. Several articles have reported that a lack of super-enhancers can lead to low expression of cancer-related genes, which profoundly affects certain oncogenic properties. Additionally, evidence suggests a link between tumorigenesis and dysregulation of signaling pathways triggered by cancer-associated chromatin enhancers. Cohen et al. highlighted the altered epigenetic features of enhancer elements as crucial drivers in the formation of human colorectal cancer [12]. Thus, targeting aberrant enhancer components has become an effective therapeutic strategy for multiple cancers [11,13]. However, substantial efforts are still needed to further elucidate the underlying mechanisms of enhancer-mediated processes in cancer and other diseases [14,15].

With the increasing integration of biology and computer science, numerous new technologies and methods have emerged in recent years, such as the application of machine learning and artificial intelligence in biological research. However, these methods also present certain challenges. Training deep learning models often requires a large amount of high-quality data, yet real biological data often contain noise, are difficult to acquire, and are often incomplete, necessitating careful selection and data cleaning. Another challenge in applying deep learning to biological research is that deep learning models are often considered black boxes, making it difficult to intuitively understand their internal decision-making processes, which is crucial for validating and inferring results in biological research. Furthermore, computational biology typically requires interdisciplinary collaboration, combining expertise from different fields to conduct research more effectively. The application and development of these new methods offer new possibilities for the identification and analysis of enhancers. Additionally, they provide new insights for disease-related research built upon high-throughput sequencing methods [16,17]. In 2015, Liu et al. proposed a prediction model that relies on only sequence data to distinguish enhancers and their strengths named iEnhancer-2L [18]. Then, Liu developed the iEnhancer-PsedeKNC [19] predictor based on a support vector machine (SVM) [20] using pseudo k-tuple nucleotide composition (PsedeKNC) to extract features from DNA sequences. On this basis, in 2018, Liu et al. proposed iEnhancer-EL [21], which is an upgraded version of iEnhancer-2L. Another effect of iEnhancer-EL is that, in addition to a dataset containing 2968 data points, an independent validation set comprising 400 data points was collected by the same method. The existence of this independent validation set benefits the evaluation of the model, making it more accurate. A convolutional neural network (CNN) [22] is typically used in image-recognition tasks, but in recent years, there have been many studies using CNN models for other tasks. Khanal and others utilized word2vec [23] to transform numerical DNA sequences into vectors, which are then input into a CNN for training. This forecasting model is named iEnhancer-CNN [24]. However, word2vec suffers from a limitation: it cannot handle long-range dependencies. Nguyen et al. proposed iEnhancer-ECNN [25], an ensemble framework based on convolutional neural networks (CNNs) that uses one-hot encodings and k-mers as the inputs. In addition, Yang et al. proposed a word-segmentation method based on statistics to extract sequence semantic information, used a generative adversarial network (GAN) [26] for data enhancement, and named the prediction model iEnhancer-GAN [27]. Although iEnhancer-GAN addresses the issue of limited data, traditional GAN models still suffer from challenges such as mode collapse, gradient vanishing, or gradient explosion, making the model training process difficult. As a kind of sequence data, DNA sequences are a good fit for sequence models such as RNNs. Aladhadh et al. proposed a model named enhancer-CNNAttGRU [28], which uses a gated recurrent unit (GRU), an upgraded version of a recurrent neural network (RNN), and combines it with an attention mechanism [29]. However, whether using an RNN [30], a GRU [31], or LSTM [32], the calculation process of these models is time-dependent, and the calculation of each time step depends on the output of the previous time step, which limits the parallelism of the model. In the latest research [33] by Bilal Ahmad Mir in October 2023, a stacked ensemble algorithm was employed to integrate six baseline classifiers. Compared to the single-model training process used in our study, this approach can be more complex. However, stacking ensemble methods typically yields better results than methods that use a single model.

To address these issues, this paper proposes a model based on the Transformer [34] encoder. The proposed model resolves the problem of parallel efficiency in traditional sequence models such as RNNs [30], GRUs [31], and LSTM networks [32]. Furthermore, by utilizing a self-attention mechanism, the Transformer can consider the contextual information of the entire input sequence and capture longer dependencies. Additionally, due to the characteristics of the Transformer model, the network depth can be increased, enabling it to perform better on large amounts of high-quality data. To replace the conventional GAN [26], this paper introduces the Wasserstein GAN with a gradient penalty (WGAN-GP) [35]. The WGAN-GP effectively expands thew datasets, resolves the data volume issues, and addresses the problems associated with GANs, such as mode collapse and gradient explosion. The combination of Transformers and the WGAN-GP allows for the effective application of our model to tasks of various data scales, thus exhibiting better transferability. Additionally, this paper introduces a coding layer designed based on the principle of k-mers, enhancing the model’s ability to capture the semantic information of DNA sequences. In Section 3, we explain the materials and optimization methods used for identifying enhancer sequences in this paper. In Section 2, the trained model is validated on an independent validation set and compared with other methods. The results indicate significant improvement in the overall performance of the proposed method on both the training dataset and the independent validation dataset. Finally, in Section 4, we discuss the conclusions of our current research.

## 2. Results and Discussion

### 2.1. Use and Nonuse of k-mers Performance

The encoding process, which determines how the input sequence is transformed into a format recognizable by the model, significantly impacts the model’s final performance. The key in this process is to fully input the information contained in the data to the model, avoiding coding issues that may result in the model receiving incomplete information, leading to deviations.

As shown in Table 1, using k-mers in the training set slightly improved the results compared to those not using k-mers. In Table 2, the accuracy of classifying enhancers and nonenhancers (task 1) on the independent validation set improved by approximately 4% when using k-mers, while the accuracy of identifying enhancer strengths (task 2) improved by almost 20% compared to not using k-mers.

By comparing the results obtained from the above-mentioned different methods, it was evident that the k-mer-based method outperformed the direct encoding of DNA sequences.

### 2.2. Sequence Generative Adversarial Network

#### 2.2.1. Effectiveness of Generative Adversarial Networks

In general, having a larger amount of available data is beneficial to the training process. However, acquiring sufficient data is not always feasible in practice. In this paper, approximately 20,000 samples were generated using the WGAN-GP on both enhancer and nonenhancer data. To validate the effectiveness of the WGAN-GP, we utilized the prediction model with and without the WGAN-GP on the training and independent validation datasets, respectively. The results are presented in Table 3 and Table 4. These tables are based on the k-mer-encoding method, and the outcomes are tabulated.

In the process of enhancer and nonenhancer classification (task 1), the training dataset was expanded by increasing the data generated by the WGAN-GP. The AUC increased from 0.795 to 0.962, and the ACC increased from 0.721 to 0.907. Improvements in the AUC, SN, SP, MCC, and ACC were also observed on the independent validation set.

In the enhancer strength prediction (task 2) phase, all metrics were significantly improved by increasing the amount of generated data in the training sets. On the training set, the SN, SP, MCC, ACC, and AUC values increased from 0.6489, 0.4911, 0.1469, 0.5687, and 0.607 to 0.9111, 0.8944, 0.8059, 0.9029, and 0.964, respectively. On the independent validation set, these values increased from 0.709, 0.482, 0.2053, 0.5955, and 0.628 to 0.978, 0.6795, 0.6892, 0.8287, and 0.9165, respectively.

By comparing the results presented in Table 3 and Table 4, it is evident that employing a GAN led to performance improvements in task 1 and task 2, as opposed to not using a GAN.

#### 2.2.2. Effectiveness of Generated Data

DNA is biological genetic information stored in a molecule that consists of four different nucleotide units. Deoxynucleotides consist of a base, deoxyribose, and a phosphate group. The bases include adenine (A), guanine (G), thymine (T), and cytosine (C). The data generated by the WGAN-GP should yield a data distribution similar to that of the real data. To visually assess the validity of the generated DNA sequences, the following figure depicts the average nucleotide composition of the actual and generated data.

**Figure 1:**
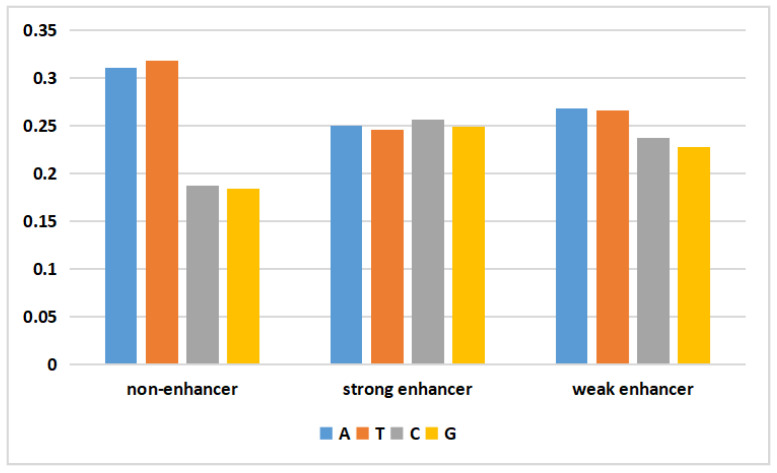
The proportion of the four types of nucleotides in the real data.

**Figure 2:**
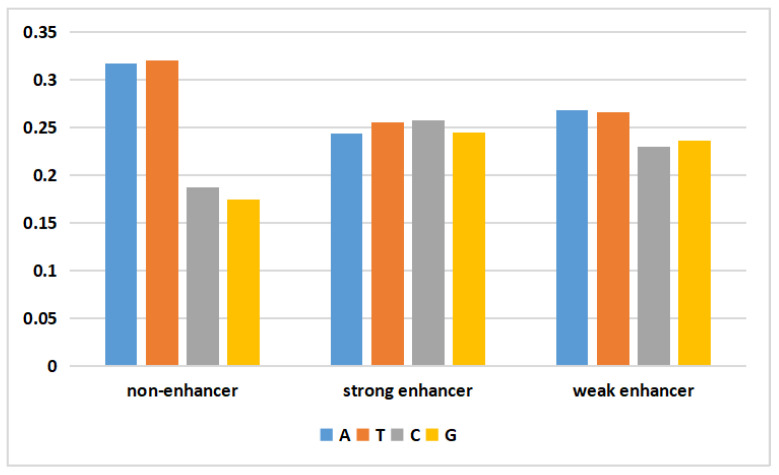
The proportion of the four types of nucleotides in the generated data.

In Figure 1, the frequencies of adenine (A) and thymine (T) in the nonenhancer region of the actual data were both approximately 0.31, while those of cytosine (C) and guanine (G) were only 0.18. In the strong enhancer, the four bases had almost the same frequency. In the weak enhancer, the frequencies of adenine (A) and thymine (T) were slightly higher than those of cytosine (C) and guanine (G). The generated data (Figure 2) exhibited a pattern similar to that of the actual data. Additionally, the difference between the mean values of the four nucleotides in the generated data and the mean values of the actual data did not exceed 3%. This result demonstrated that the WGAN-GP effectively learned and simulated the nucleotide distribution characteristics of real DNA sequences, thereby generating artificial enhancer sequences with similar statistical properties. This similarity plays a positive role in generating artificial DNA sequences that possess biological significance and functionality.

Based on the results of both experiments, it can be concluded that utilizing the WGAN-GP to supplement experimental data enhanced the performance of our model in two tasks: classifying enhancers and nonenhancers and predicting the enhancer strength.

### 2.3. Results

In the classification of enhancers and nonenhancers (task 1), we treated strong and weak enhancers as positive samples and nonenhancers as negative samples. Subsequently, we trained the WGAN-GP for strong enhancers, weak enhancers, and nonenhancers. The generated samples of strong enhancers, weak enhancers, and nonenhancers were added to the original training dataset. The ratio of positive samples (including strong and weak enhancers in 1:1) to negative samples (nonenhancers) was 1:1.

In the enhancer strength prediction (task 2), we treated strong enhancers as positive samples and weak enhancers as negative samples. Similar to task 1, we added the generated strong enhancers and weak enhancers to the original dataset for training, maintaining a ratio of 1:1.

**Figure 3:**
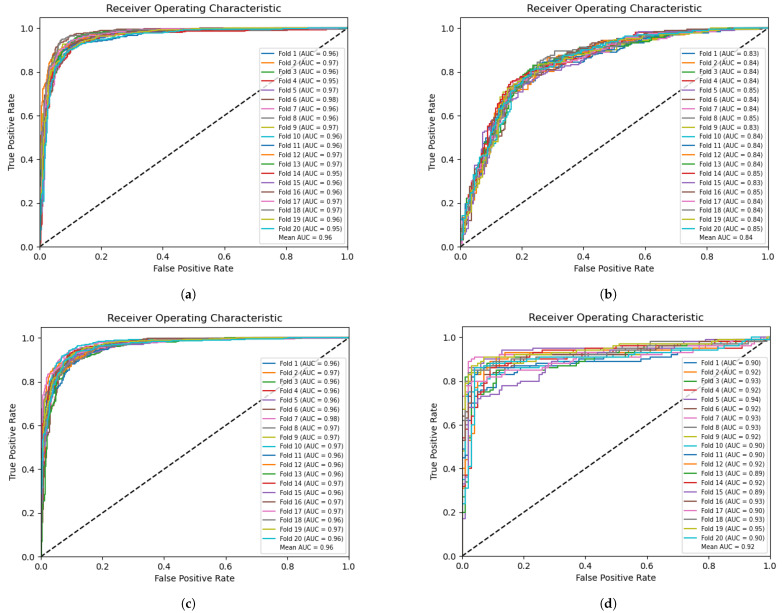
The ROC curves for the classification of enhancers and nonenhancers in the training set are plotted in (**a**). The ROC curves for the classification of enhancers and nonenhancers in the independent validation set are plotted in (**b**). The ROC curves of enhancer strength recognition on the training set are plotted in (**c**). The ROC curves of enhancer strength recognition on the independent validation set are plotted in (**d**).

To evaluate the performance, we conducted 20-fold cross-validation on the dataset. Figure 3a and Figure 3c illustrate the classification (task 1) and prediction (task 2) receiver operating characteristic (ROC) curves on the training set, respectively. An average AUC of 0.96 was achieved for both tasks. Figure 3b presents the results of classifying enhancers and nonenhancers on the independent validation set (task 1), with an average AUC of 0.84. Figure 3d demonstrates the prediction of enhancer strength on the independent validation set (task 2), with an average AUC of 0.92.

**Figure 4:**
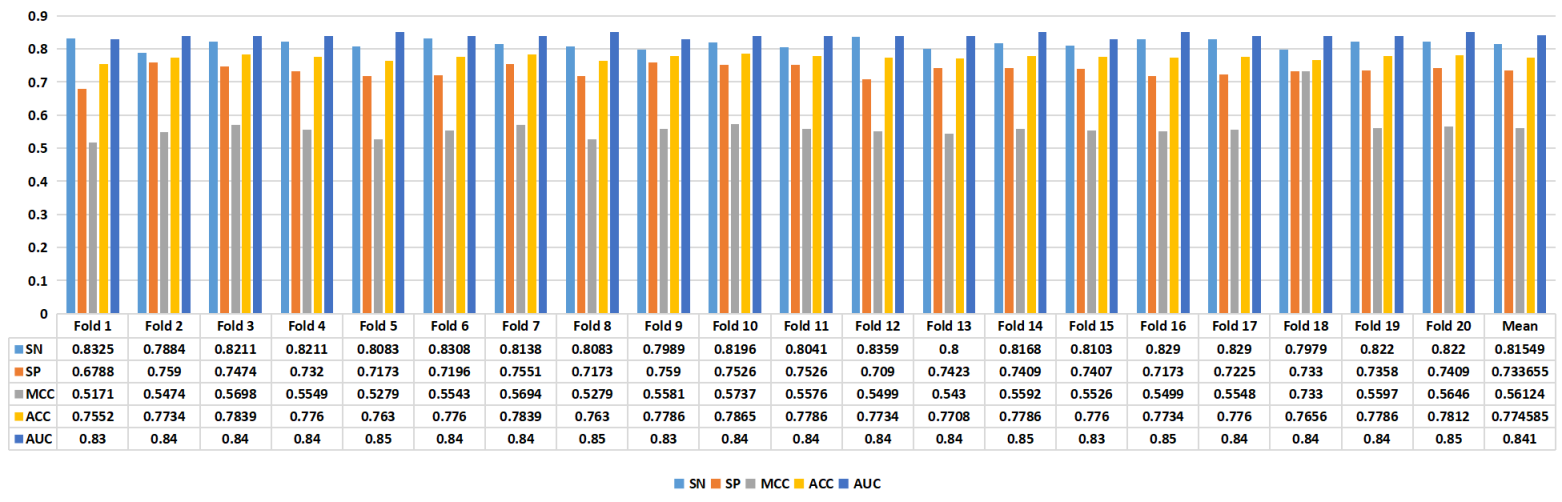
The performance of the proposed model using the benchmark enhancer sequence datasets (task 1).

**Figure 5:**
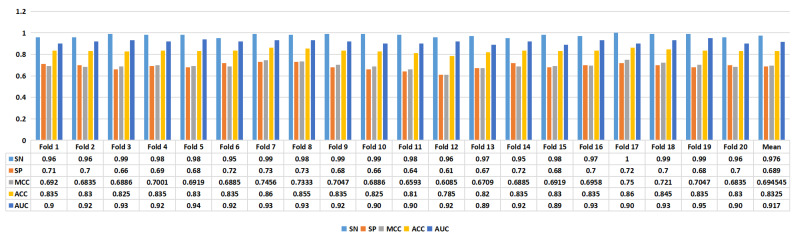
The performance of the proposed model using the benchmark enhancer sequence datasets (task 2).

Figure 4 presents the experimental results of the proposed model. In task 1, we achieved an average ACC of 0.7746, while in task 2, we achieved an average ACC of 0.8325. Furthermore, in task 1, the model achieved an SN of 0.8155, SP of 0.7337, MCC of 0.5612, ACC of 0.7746, and AUC of 0.841. In task 2, the proposed model’s performance metrics were as follows: SN of 0.976, SP of 0.689, MCC of 0.6945, ACC of 0.8325, and AUC of 0.917, as depicted in Figure 5. The analysis of the results showed that the model performed better on task 1 than on task 2, suggesting that distinguishing enhancers from nonenhancers is easier than predicting enhancer strength.

### 2.4. Comparisons with Existing Methods

The performance of the proposed method was then compared with that of existing methods, including iEnhancer-2L [18], iEnhancer-EL [21], iEnhancer-ECNN [25], iEnhancer-CNN [24], iEnhancer-GAN [27], enhancer-CNNAttGRU [28], and iEnhancer-PsedeKNC [19].

The iEnhancer-2L method is a two-layer classifier that utilizes the first layer to determine whether a sequence is an enhancer and the second layer to determine the strength of the sequence. Both iEnhancer-2L and iEnhancer-EL utilize a training set consisting of 2968 data entries and an independent validation set of 400 data entries. The iEnhancer-CNN and iEnhancer-ECNN models primarily utilize CNNs, which are commonly used in image recognition, and CNNs have been applied to other tasks in recent years. However, CNNs have limited capabilities in capturing global features from the data. Due to data limitations, these models fail to uncover deeper dependencies among data. As DNA sequences are complex sequential data, their features may require operations beyond convolutions for effective learning. iEnhancer-CNN employs word2vec for processing DNA sequences, while Enhancer-CNNAttGRU uses a GRU, which is a special type of sequential model. However, both word2vec and GRUs struggle with handling long-range dependencies, and the fact that each time step of the model relies on the output of the previous time step results in low parallel efficiency. Although iEnhancer-GAN addresses the issue of insufficient data points, traditional GANs suffer from problems such as mode collapse, vanishing gradients, and exploding gradients, making it difficult for the model to converge during training and leading to relatively poor data generation. To address the aforementioned issues with sequential models, our research incorporated a Transformer. The Transformer resolves the parallel efficiency problem in traditional sequential models and, as the most-advanced model in this field, employs self-attention mechanisms to process information across the entire sequence, enabling it to capture long-range dependencies. Additionally, we replaced the original GAN with the WGAN-GP to generate more high-quality data for model training, overcoming the training difficulties associated with GANs. However, the WGAN-GP can only amplify data features, and it cannot enhance data diversity, which is one limitation of our research. Due to the characteristics of the Transformer and the integration of the WGAN-GP for data generation, our research can be flexibly applied to tasks of various data scales, demonstrating good transferability.

**Figure 6:**
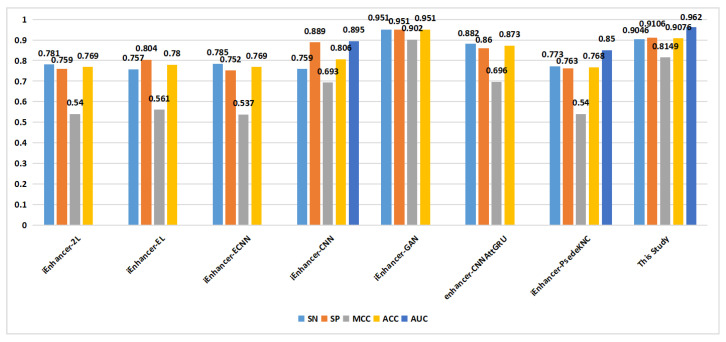
Comparison of proposed method with other methods on the training set in enhancer and nonenhancer classification (task 1).

**Figure 7:**
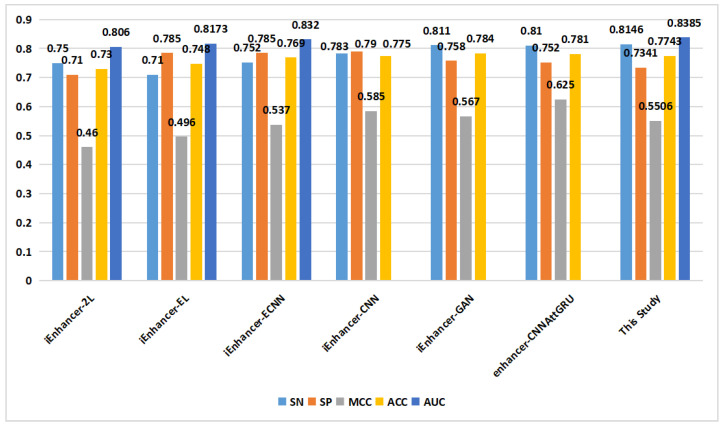
Comparison of proposed method with other methods on the independent validation set in enhancer and nonenhancer classification (task 1).

As shown in Figure 6, in the classification of enhancers and nonenhancers (task 1), apart from the proposed method, only one method achieved an ACC of 0.9 on the training set. On the independent validation set (Figure 7), the proposed method outperformed the other methods in terms of the SN metric, reaching a value of 0.815. Additionally, the proposed method achieved a similar level of the ACC compared to the that of the other methods.

**Figure 8:**
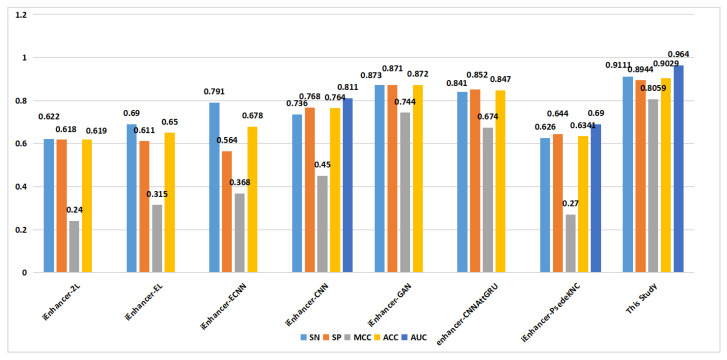
Comparison of proposed method with other methods on the training set in enhancer strength identification (task 2).

**Figure 9:**
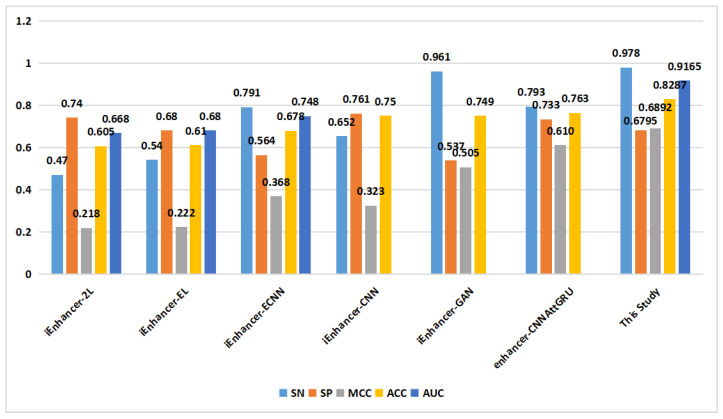
Comparison of proposed method with other methods on the independent validation set in enhancer strength identification (task 2).

In the prediction of enhancer strength (task 2), as depicted in Figure 8, the proposed method demonstrated excellent performance in terms of the SN, SP, MCC, ACC, and AUC on the training set. On the independent validation set (Figure 9), the proposed method achieved an ACC of 0.829, surpassing that of the other methods.

All the comparison results were obtained from the same dataset, which consisted of only 2968 data points. Such a small-scale dataset fails to meet the basic requirements for an effective model. In the field of computer vision image recognition, traditional data augmentation methods such as scaling, panning, and flipping can be applied to images. However, DNA sequence data lack conditions similar to images, which is the primary reason we utilized a GAN to generate the data.

## 3. Materials and Methods

### 3.1. Benchmark Datasets

The dataset in this paper comprises two parts: a training set and an independent validation set. The training set was trained using the basic dataset constructed by Liu et al. [18] and is also utilized in iEnhancer-2L [18], iEnhancer-EL [21], iEnhancer-ECNN [25], iEnhancer-CNN [24], iEnhancer-GAN [27], enhancer-CNNAttGRU [28], iEnhancer- PsedeKNC [19], and other experiments. According to the information on the chromatin state of nine cell lines, using the CD-HIT software [36], the benchmark dataset was constructed by extracting DNA fragments with a length of 200 bp and removing fragment pairs with a sequence identity greater than 20%. After randomly selecting nonenhancers and weak enhancers based on human embryonic stem cells, the training dataset included 1484 enhancers (742 strong enhancers and 742 weak enhancers) and 1484 nonenhancers.

The independent validation set [21] used the same method of collectingthe training set, including 200 enhancers (100 of the 100 strong enhancers and weak enhancers) and 200 nonenhancers. For a fair comparison with existing methods, the validation process of this experiment was conducted on this independent validation set.

In the classification stage of enhancers and nonenhancers (first task), both positive samples (strong and weak enhancers) and negative samples (nonenhancers) were used for training and testing. In the enhancer strength prediction stage (second task), positive samples (strong enhancers) and negative samples (weak enhancers) were utilized for training and testing. k-fold cross-validation was also employed during the training process. After augmenting the training data with the WGAN-GP, the training set was divided into 20 folds, with 19 folds used for training and the remaining fold used for testing. After training, each model was verified on an independent validation set.

### 3.2. k-mer Coding

Establishing the relationship between biological language and natural language is an important issue that often directly impacts the outcome of the model. To address this, we divided the DNA sequence into continuous k-bases and encoded them using the principle of k-mers, which is a concept in genomics and bioinformatics commonly used to represent k consecutive bases in a DNA or ribonucleic acid (RNA) sequence. Bases can include adenine (A), thymine (T), guanine (G), and cytosine (C).

**Figure 10:**
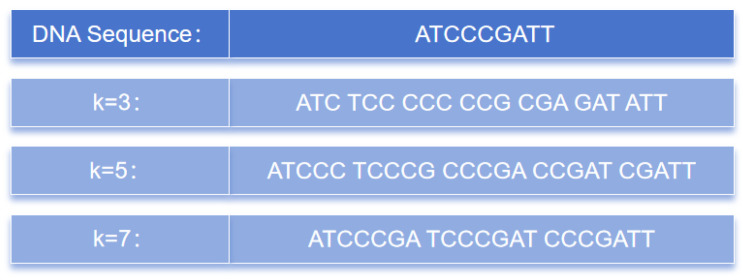
k-mers at different k values.

The k-mer method is one of the most-commonly used analytical approaches in genomics and bioinformatics. It is simple, efficient, and capable of preserving the local information of DNA sequences, thereby capturing important features. Furthermore, k-mers are widely used and can be applied to various areas, such as similarity comparison, sequence classification, and pattern recognition in DNA sequences. As a result, they have been widely employed in the data analysis of genomics and bioinformatics and are becoming some of the most-prevalent analytical methods in these fields. Figure 10 illustrates the partitioning of consecutive k nucleotides into different values of the k-mers. From a sequence of length L, a total of L-k+1 k-mer sequences can be obtained.

### 3.3. Wasserstein GAN with a Gradient Penalty

In our study, we utilized the dataset provided by Liu et al. [18], which consists of 2968 instances. Due to the limited data volume, it is challenging for the model to effectively learn underlying features and patterns within the data. Additionally, a larger amount of data can help mitigate overfitting and improve the model’s generalization ability, enabling it to adapt better to unseen new data. Hence, we conducted supplementary training using a WGAN-GP model to generate additional data, aiming to enhance the performance of the final model.

The WGAN-GP, an improved GAN that aims to address some of the issues in traditional GANs, such as training instability and mode collapse, is based on the Wasserstein distance [37]. This distance measures the disparity between the distributions of real and generated data. The conventional objective functions, Jensen–Shannon (JS) divergence and Kullback–Leibler (KL) divergence, used in traditional GANs exhibit abrupt changes. In high-dimensional spaces, if two distributions have no overlapping regions, the KL and JS divergence functions fail to provide any meaningful gradient information, and this is a significant challenge in deep learning. The Wasserstein distance was introduced to address this issue. The Wasserstein distance possesses better mathematical properties and gradient derivability, contributing to a more-stable training process. For two distributions, *P* and *Q*, the Wasserstein distance can be expressed as:

$$W\left(P,Q\right)=\underset{\gamma \in \Pi (P,Q)}{\inf}E_{(x,y)∼\gamma }[|x-y|]$$

**Figure 11:**
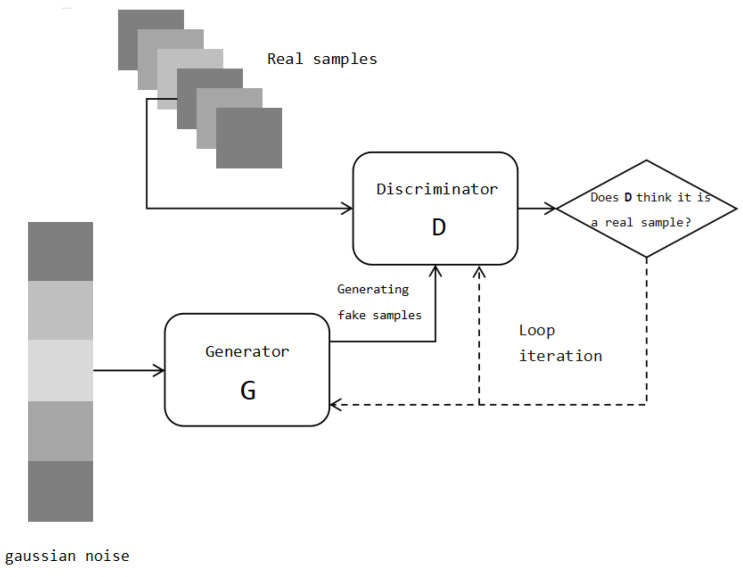
Generative adversarial network architecture.

Additionally, the WGAN-GP introduces a gradient penalty to optimize the Wasserstein distance. The purpose of the gradient penalty is to constrain the gradient of the discriminator during training to ensure that the discriminator satisfies the Lipschitz continuity condition. In this way, gradient explosion or disappearance during model training can be avoided, and the stability of training can be further improved. As shown in Figure 11, a GAN includes a generator and a discriminator. The generator input is a random noise vector, which is mapped to the data space to generate synthetic samples. The discriminator receives real samples and synthetic samples produced by the generator as the input and attempts to distinguish between them. Unlike the binary discriminator of a GAN, the output of the discriminator in the WGAN-GP is used to measure the distance between the generated sample and the true sample.

In our research, we chose a single-layer ResNet model as the main architecture for the generator and discriminator in WGAN-GP. This was due to the limited availability of real training data; that is, only 2968 instances were available. Using a deeper architecture would increase the risk of overfitting and hinder the model’s generalization ability. For the size of each layer, we experimented with several values, including 128, 256, and 512. Keeping other variables constant, we found that a hidden layer size of 256 yielded the best performance.

### 3.4. Construction of Neural Network

The Transformer [34] is a deep learning model used for natural language processing (NLP) tasks [38], which was proposed by Vaswani et al. in 2017 and has been widely adopted in various NLP applications, including text classification, named entity recognition, and question-answering systems. Traditional sequence models such as RNNs [30] encounter issues such as vanishing and exploding gradients when handling long sequences. The Transformer model addresses these problems by introducing a self-attention mechanism, which enables the model to process positional information across the input sequence in parallel.

As a special type of sequence data, DNA sequences exhibit certain correlations and dependencies. The Transformer, as a sequence model, effectively learns and models these relationships to extract important features from DNA sequences. Moreover, DNA sequences typically have long lengths, and traditional sequence models such as RNNs encounter computational inefficiency issues when handling such long sequences. The Transformer model efficiently addresses this problem by utilizing a self-attention mechanism to compute the features in parallel, which is particularly crucial for improving the model performance in DNA-sequence-related tasks. Furthermore, the general adaptability of the Transformer model enables its seamless application in various types of DNA sequence tasks. In summary, the Transformer model offers a powerful and versatile tool for DNA sequence analysis.

**Figure 12:**
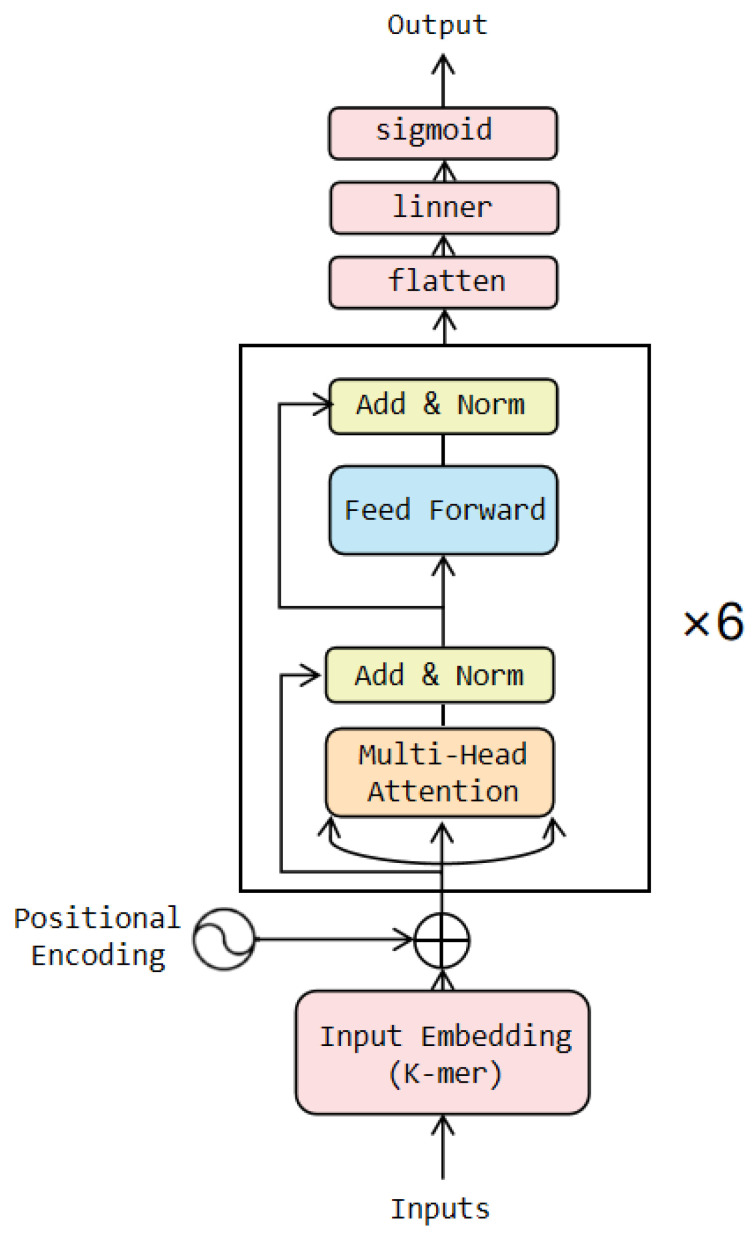
Network architecture.

The Transformer consists of a group of encoders and decoders. The encoder encodes the input sequence into a series of high-dimensional representations, while the decoder generates the target sequence based on the output of the encoder. In this study, the WGAN-GP model was initially trained using the training set, then a portion of the data was generated using the WGAN-GP and added to the training set to augment it. Subsequently, the expanded training set was fed into the classification model for further training. In this paper, we utilized only the encoder part of the Transformer model in the classification model, and the architecture is illustrated in Figure 12. The coding layer designs the coding method based on the principle of k-mers and incorporates positional information into the encoded vector. After passing through six Transformer encoder blocks, the one-dimensional vector is transformed into a multidimensional vector. Finally, in the output layer, the multidimensional vector is dimensionally reduced and compressed within the range of [0, 1] [39]. Finally, the vector is then mapped to the corresponding class label through a threshold decision.

In our model, we utilized a 6-layer Transformer encoder. This decision was driven by the fact that we augmented our 2968 instances of training data with 20,000 samples generated using the WGAN-GP. A smaller number of layers would make it difficult to capture the true distribution of the data. For other parameters, we employed a grid search strategy to identify the optimal parameter combinations and found that the model’s performance was maximized when the hidden layer size was 512, 5-mer was applied, and a weight decay parameter value of 0.01 was used.

### 3.5. Performance Measures

In this study, we utilized 20-fold cross-validation to evaluate the performance of the enhancer classifier and enhancer strength predictor. Specifically, the training dataset was randomly divided into 20 roughly equal and disjointed subsets. Each subset was used as a test set in turn, while the remaining subsets were merged to train the predictor. This process involved training 20 independent models simultaneously and evaluating them on the respective test sets. Furthermore, after the final model was trained, a comprehensive performance evaluation was conducted on an independent validation set.

To evaluate the performance of these models, we used commonly used metrics such as sensitivity (*SN*), specificity (SP), accuracy (*ACC*), Matthews’ correlation coefficient (*MCC*), and receiver operating characteristic (*ROC*) curves. These metrics are defined as follows:

(1) $$SN=\frac{TP}{TP+FN}$$

(2) $$SP=\frac{TN}{TN+FP}$$

(3) $$ACC=\frac{TP+TN}{TP+TN+FP+FN}$$

(4) $$MCC=\frac{TP\times TN-FP\times FN}{\sqrt{\left(TP+FP)(TP+FN)(TN+FP)(TN+FN\right)}}$$

### 3.6. Experimental Method

We conducted three experiments to demonstrate the effectiveness of the proposed model. In the first experiment, we modified only the coding method and compared the results of using k-mers to those without using k-mers. The second experiment demonstrated the effectiveness of the data generated by using the WGAN-GP through two aspects: comparing the results with and without using the GAN and comparing the proportion of four nucleotides between the real data and the generated data. In the third experiment, we verified the model using both k-fold cross-validation and independent validation methods and compared the results with those obtained from other methods.

All the experimental processes were related to two tasks, namely the classification of enhancers and nonenhancers (task 1) and the identification of strength enhancers (task 2).

## 4. Conclusions

Enhancers are DNA sequences located in the upstream or downstream area of the genome and play an important role in regulating the transcription activity of adjacent genes by binding to transcription factors. Identifying enhancers and their strengths is crucial for gaining insight into gene regulatory networks, predicting gene functions, studying disease associations, and providing potential targets and strategies for genome editing and gene therapy. These studies contribute to our understanding of gene expression regulation and provide valuable information and tools for biomedical research and applications.

As real data are limited, we trained a generative model based on the WGAN-GP to generate additional data. Generating partial data increases the possibility of capturing the data characteristics of subsequent classification models. Based on expanding the dataset, a classification model was designed based on the Transformer encoder. The Transformer is a powerful sequence model with high parallelism, which can effectively handle long-distance dependencies. Compared with existing technologies, the model proved to be powerful, valuable, and efficient. In future work, we will focus on feature extraction from the data, enabling its application in research related to enhancer-associated disease mechanisms and assisting in drug development.

## Figures and Tables

**Table 1:** The metrics on the training set.

	SN	SP	MCC	ACC	AUC
without k-mers (task 1)	0.8929	0.9113	0.8054	0.9026	0.9565
with k-mers (task 1)	0.9046	0.9106	0.8149	0.9076	0.9620
without k-mers (task 2)	0.8046	0.8131	0.6189	0.8095	0.8895
with k-mers (task 2)	0.9111	0.8944	0.8059	0.9029	0.964

**Table 2:** The metrics on the independent validation set.

	SN	SP	MCC	ACC	AUC
without k-mers (task 1)	0.7454	0.7497	0.4954	0.7475	0.81
with k-mers (task 1)	0.8146	0.7341	0.5506	0.7743	0.8385
without k-mers (task 2)	0.6950	0.5370	0.2358	0.6160	0.666
with k-mers (task 2)	0.978	0.6795	0.6892	0.8287	0.9165

**Table 3:** The metrics on the training set.

	SN	SP	MCC	ACC	AUC
without GAN( task 1)	0.6536	0.787	0.4478	0.721	0.795
with GAN (task 1)	0.9046	0.9106	0.8149	0.9076	0.962
without GAN (task 2)	0.6489	0.4911	0.1469	0.5687	0.607
with GAN (task 2)	0.9111	0.8944	0.8059	0.9029	0.964

**Table 4:** The metrics on the independent validation set.

	SN	SP	MCC	ACC	AUC
without GAN (task 1)	0.7891	0.7291	0.5222	0.7591	0.837
with GAN (task 1)	0.8146	0.7341	0.5506	0.7743	0.8385
without GAN (task 2)	0.709	0.482	0.2053	0.5955	0.628
with GAN (task 2)	0.978	0.6795	0.6892	0.8287	0.9165

## Data Availability

Data is contained within the article. Our source code is available at https://github.com/TianyuFeng1110/iEnhancer-TransformerEncoder.

## Abbreviations

The following abbreviations are used in this manuscript:

CNN	convolutional neural network
ECNN	ensemble of CNNs
LSTM	long short-term memory
GRU	gated recurrent unit
NLP	natural language processing
ACC	accuracy
SN	sensitivity
SP	specificity
MCC	Matthews’ correlation coefficient
PseKNC	pseudo k-tuple nucleotide composition
ROC	receiver operating characteristic
SVM	support vector machine
GAN	generative adversarial network
WGAN-GP	Wasserstein generative adversarial network with a gradient penalty
AUC	area under the ROC curve
